# Trends in Low-Value Carotid Imaging in the Veterans Health Administration From 2007 to 2016

**DOI:** 10.1001/jamanetworkopen.2020.15250

**Published:** 2020-09-04

**Authors:** Timothy S. Anderson, Samuel Leonard, Alysandra J. Zhang, Erin Madden, Danielle Mowery, Wendy W. Chapman, Salomeh Keyhani

**Affiliations:** 1 Division of General Medicine, Beth Israel Deaconess Medical Center, Boston, Massachusetts; 2 Richard A. and Susan F. Smith Center for Outcomes Research in Cardiology, Beth Israel Deaconess Medical Center, Boston, Massachusetts; 3 Northern California Institute of Research and Education, San Francisco; 4 Department of Counseling and Clinical Psychology, Teachers College, Columbia University, New York, New York; 5 Department of Bioinformatics, Epidemiology, and Informatics, University of Pennsylvania, Philadelphia; 6 University of Melbourne, Melbourne, Australia; 7 Division of General Internal Medicine, Department of Medicine, University of California, San Francisco; 8 San Francisco Veterans Affairs Medical Center, San Francisco, California

## Abstract

**Importance:**

As part of the Choosing Wisely campaign, primary care, surgery, and neurology societies have identified carotid imaging ordered for screening, preoperative evaluation, and syncope as frequently low value.

**Objective:**

To determine the changes in overall and indication-specific rates of carotid imaging following Choosing Wisely recommendations.

**Design, Setting, and Participants:**

This serial cross-sectional study compared annual rates of carotid imaging before Choosing Wisely recommendations (ie, 2007 to 2012) and after (ie, 2013 to 2016) among adults receiving care in the Veterans Health Administration (VHA) national health system. Data analysis was performed from April 10, 2019, to November 27, 2019.

**Exposures:**

Release of the Choosing Wisely recommendations.

**Main Outcomes and Measures:**

Annual rates of overall imaging, imaging ordered for stroke workup, imaging ordered for low-value indications (ie, screening owing to carotid bruit, preoperative evaluation, and syncope). Indications were identified using a text lexicon algorithm based on electronic health record review of a stratified random sample of 1000 free-text imaging orders. The subsequent performance of carotid procedures within 6 months after carotid imaging was assessed.

**Results:**

Between 2007 and 2016, 809 071 carotid imaging examinations were identified (mean [SD] age of patients undergoing imaging, 69 [10] years; 776 632 [96%] men), of which 201 467 images (24.9%) were ordered for low-value indications (67 064 [8.2%] for carotid bruit, 25 032 [3.1%] for preoperative evaluation, and 109 400 [13.5%] for syncope), 257 369 (31.8%) for stroke workup, and 350 235 (43.3%) for other indications. Imaging for carotid bruits declined across the study period while there was no significant change in imaging for syncope or preoperative evaluation. Compared with the 6 years before, during the 4 years following Choosing Wisely recommendations, there was no change in the trend for syncope, a small decline in preoperative imaging (post–Choosing Wisely trend, −0.1 [95% CI, −0.1 to <−0.1] images per 10 000 veterans), and a continued but less steep decline in imaging for carotid bruits (post–Choosing Wisely trend, −0.3 [95% CI, −0.3 to −0.2] images per 10 000 veterans). During the study period, 17 689 carotid procedures were identified, of which 3232 (18.3%) were preceded by carotid imaging ordered for low-value indications.

**Conclusions and Relevance:**

These findings suggest that Choosing Wisely recommendations were not associated with a meaningful change in low-value carotid imaging in a national integrated health system. To reduce low-value testing and utilization cascades, interventions targeting ordering clinicians are needed to augment the impact of public awareness campaigns.

## Key Points

**Question:**

How has utilization of low-value carotid imaging for carotid bruits, preoperative evaluation, and syncope changed in the Veterans Health Administration system from 2007 to 2016?

**Findings:**

In this serial cross-sectional study of 809 071 carotid imaging examinations in the national Veterans Affairs Health system, there was a modest decrease in rates of imaging for carotid bruits that predated Choosing Wisely recommendations, and no significant trends in rates of preoperative carotid imaging or imaging for syncope.

**Meaning:**

These findings suggest that to reduce low-value testing and utilization cascades, interventions targeting ordering clinicians are needed to augment the impact of public awareness campaigns.

## Teaser

This serial cross-sectional study examines rates of low-value carotid imaging orders in the Veterans Health Administration before and after the release of the Choosing Wisely campaign.

## Introduction

Overuse of diagnostic testing is a recognized problem in the United States,^1^ which leads to unnecessary spending and has the potential to trigger health care utilization cascades leading to direct patient harms.^2,3^ The Choosing Wisely campaign, launched in 2012 by the ABIM Foundation and Consumer Reports, is a physician-driven effort to foster conversations around overuse through the creation of lists of potentially unnecessary testing by medical professional societies.^4^ Although Choosing Wisely has received significant attention from physicians,^5,6^ concern has been raised that professional society lists often include tests performed infrequently and focus on services typically ordered or performed by other specialties.^6,7,8,9^ To date, most research on outcomes associated with Choosing Wisely campaigns has been limited to the first year of Choosing Wisely implementation and has not evaluated downstream utilization cascades.^10,11,12^

One frequently identified low-value test is carotid imaging. The role of carotid imaging in the evaluation of symptomatic patients who have stroke or transient ischemia attacks (TIA) is well established, as a substantial proportion of ischemic strokes are attributable to carotid atherosclerosis.^13^ Carotid imaging is performed for many indications other than stroke evaluation, the appropriateness of which are often uncertain.^14^ When patients who received carotid imaging for uncertain indications are found to have carotid stenosis, they may be offered a revascularization procedure for primary stroke prevention, exposing them to periprocedural risk of stroke, death, and myocardial infarction despite evolving evidence that suggests a potential lack of benefit.^15,16^ As a result, carotid imaging has been a target of multiple Choosing Wisely recommendations. In February 2013, the American Academy of Family Physicians^17^ recommended against screening for carotid artery stenosis in asymptomatic adults, building on prior recommendations against screening by the US Preventative Services Taskforce (USPSTF) released 2007 and updated in 2014.^18^ Also in 2013, the American Academy of Neurology^19^ recommended against carotid imaging for syncope without other neurologic symptoms, and the Society of Thoracic Surgeons^20^ recommended against preoperative carotid imaging prior to cardiac surgery in asymptomatic patients. To our knowledge, the proportion of imaging conducted for low-value indications, as opposed to evidence-based stroke workup, have not been described, and the associations of professional society recommendations with carotid imaging tests are unknown.

To understand the association of Choosing Wisely recommendations with carotid imaging tests, we examined national trends in carotid imaging in the Veterans Health Administration (VHA) health system over a 10-year period from 2007 to 2016. We conducted a time-series analysis comparing rates of carotid imaging before and after Choosing Wisely recommendations were released. To determine the cascade of utilization following low-value carotid imaging, we also examined the number of images ordered for low-value indications that were followed by a carotid revascularization procedure.

## Methods

### Study Design and Data Source

We conducted a serial cross-sectional study of carotid imaging and interventions performed in the VHA health system between January 1, 2007, and December 31, 2016. Demographic, comorbidity, and imaging data were obtained from the VHA Clinical Data Warehouse. This study was approved by the University of California, San Francisco, institutional review board. This study was restricted to secondary data analysis; thus, the requirement for informed consent from participants was waived by the University of California, San Francisco. Data analysis was performed from April 10, 2019, to November 27, 2019. This study followed the Strengthening the Reporting of Observational Studies in Epidemiology (STROBE) reporting guideline.

### Identifying Carotid Images

We identified all imaging tests performed by VHA radiology departments to assess for carotid stenosis, including carotid ultrasonography, magnetic resonance angiography, and computed tomographic angiography. As procedure codes for carotid imaging are nonspecific and may include other head and neck imaging, we used a previously developed natural language processing algorithm to identify images assessing carotid stenosis.^21^

### Identifying Indications for Imaging

We sought to identify carotid images ordered for low-value indications (ie, syncope, screening, and preoperative testing) and those ordered for symptomatic indications (ie, stroke, TIA, or related neurologic symptoms) for which carotid imaging is a part of standard care. Prior studies of low-value testing have relied on diagnosis and procedure codes within administrative claims to identify indications.^10,22,23^ This approach has substantial risk of misclassification because the diagnosis resulting from test rather than the initial indication for ordering the test may have been recorded. For testing related to signs and symptoms, such as syncope, diagnoses codes may lack sensitivity.^24^ Furthermore, prior studies of preoperative testing have often identified testing based on a specified time period (eg, 30 days) prior to a surgical procedure. This approach may misclassify testing performed for indications other than preoperative testing during the specified time period and may miss preoperative testing performed for a planned procedure that did not ultimately occur.^22,25,26^

To overcome the limitations of prior approaches, we developed a text lexicon algorithm that searched documentation entered into Study Reason and Clinical History free-text fields by clinicians at the time of test ordering. The lexicon was developed iteratively through electronic health record review of a stratified random sample of 1000 carotid images (eAppendix in the Supplement).

The final lexicon was then applied to the full cohort to categorize each image indication as stroke workup, syncope, bruit, preoperative evaluation, or other. The stroke workup category included images ordered for a stated indication of stroke or TIA and images ordered for neurologic symptoms that may be related to stroke. Consistent with prior studies, imaging for syncope included imaging related to presyncope, dizziness, lightheadedness, and orthostatic hypotension but excluded indications that noted bilateral or unilateral neurologic symptoms that may be related to stroke.^15^ We chose to focus on tests ordered to evaluate carotid bruits as a proxy for screening, as auscultation of the carotids for bruit is a common but clinically unproven initial screening test,^27^ which often leads to carotid imaging and is recommended against by the USPSTF.^18^

We found that the text lexicon had a sensitivity of 78% to 95% and specificity of 91% to 99% across indications (eAppendix in the Supplement). As carotid imaging is an evidence-based part of a stroke workup, we sought to be conservative and classified images identified by the lexicon as part of a stroke or TIA workup and all other images ordered in the 6 months following a clinically documented stroke or TIA as part of a stroke or TIA workup.

### Carotid Revascularization Procedures

For each carotid image, we determined whether or not a carotid procedure was performed within the subsequent 6 months. Carotid revascularization procedures, including carotid endarterectomy and carotid artery stenting, were identified using *International Classification of Diseases, Ninth Revision,*^28^ *International Statistical Classification of Diseases and Related Health Problems, Tenth Revision*,^29^ and *Current Procedural Terminology* codes (eTable in the Supplement). If additional images were obtained within 6 months, we included only the initial image and indication, as follow-up imaging is typically obtained in preparation for surgery rather than for a new indication (eg, obtaining magnetic resonance angiography for surgical planning following an initial ultrasonograph). Once a patient underwent a carotid intervention, we excluded all subsequent images and procedures from analysis, as these events most often represent 2-stage interventions for bilateral stenosis or management of carotid restenosis.

### Statistical Analysis

We conducted longitudinal analyses using segmented time series. First, we present descriptive characteristics of patients receiving imaging. We then present the annual number of carotid images performed overall and by image indication.

We determined the annual rate of carotid imaging (standardized per 10 000 veterans receiving care in the VHA health system) overall, for low-value indications (ie, syncope, bruit, and preoperative) and for stroke workup. Outcomes were inspected graphically and ordinary least-squares regressions were fit with Newey-West SEs to account for autocorrelation.^30^ We assessed for a significant trend in annual imaging rates over the entire study period using the Mann-Kendall nonparametric test. We then conducted times series analysis with preintervention and postintervention periods determined by the release of the Choosing Wisely recommendations on low-value carotid imaging in February 2013.^17,19,20^ The preintervention period included all testing performed between January 1, 2007, and December 31, 2012. The postintervention period included all testing performed between January 1, 2013, and December 31, 2016. Preintervention and postintervention trends (slopes), the immediate level change in 2013, and the difference in slopes between preintervention and postintervention trends are reported as annual images per 10 000 veterans with 95% CIs.

We lastly present the number and proportion of carotid images that were followed by a carotid procedure, overall and by image indication. Statistical analyses were conducted using Stata statistical software version 16 (StataCorp). *P* values were 2-sided, and statistical significance was set at .05.

## Results

### Patient Characteristics

During the study period, 809 071 carotid images were performed by the VHA radiology service. The mean age (SD) of patients undergoing imaging was 68.6 (10.1) years, and 776 632 patients were men (96.0%). Comorbidities were common, including hypertension (629 206 patients [77.8%]), coronary artery disease (303 214 patients [37.5%]), and prior stroke (125 266 patients [15.5%]), and 253 854 patients (33.6%) were current smokers (Table 1).

**Table 1.** Characteristics of Patients Who Underwent Carotid Imaging Between 2007 and 2016

Characteristic	Patients, No. (%)		
	Full cohort (N = 809 170)	Before Choosing Wisely (n = 474 819)	Following Choosing Wisely (n = 334 252)
Age, mean (SD), y	67.6 (10.0)	67.2 (10.2)	68.0 (9.6)
Sex			
Men	776 632 (96.0)	457 641 (96.4)	318 991 (95.4)
Women	25 644 (3.2)	13 558 (2.9)	12 086 (3.6)
Unknown	6795 (0.8)	3620 (0.8)	3175 (0.9)
Race/ethnicity			
White	630 491 (77.9)	370 283 (78.0)	260 208 (77.8)
Black	74 302 (9.2)	40 532 (8.5)	33 770 (10.1)
Hispanic	34 337 (4.2)	19 384 (4.1)	14 953 (4.5)
Other	11 848 (1.5)	6689 (1.4)	5159 (1.5)
Unknown	58 093 (7.2)	37 931 (8.0)	20 162 (4.2)
Comorbidities			
Atrial fibrillation	80 256 (9.9)	43 591 (9.2)	36 665 (11)
Chronic kidney disease	206 794 (25.6)	113 876 (24.0)	92 918 (27.8)
Cirrhosis	20 997 (2.6)	9845 (2.1)	11 152 (3.3)
Congestive heart failure	81 209 (10.0)	46 322 (9.8)	34 887 (10.4)
Coronary artery disease	303 214 (37.5)	181 374 (38.2)	121 840 (36.5)
Diabetes	317 177 (39.2)	181 392 (38.2)	135 785 (40.6)
Hyperlipidemia	568 596 (70.3)	335 331 (70.6)	233 265 (69.8)
Hypertension	629 206 (77.8)	370 793 (78.1)	258 413 (77.3)
Peripheral vascular disease	134 589 (16.6)	80 229 (16.9)	54 360 (16.3)
Stroke	125 266 (15.5)	63 225 (13.3)	62 041 (18.6)
Smoking status			
Current	253 854 (31.4)	149 502 (31.4)	104 532 (31.3)
Former	283 788 (35.1)	163 378 (34.4)	120 410 (36.0)
Never	177 681 (22.0)	99 401 (20.9)	78 280 (23.4)
Unknown	93 568 (11.6)	62 538 (13.7)	31 030 (9.2)

### Indications for Imaging

**Figure 1.**
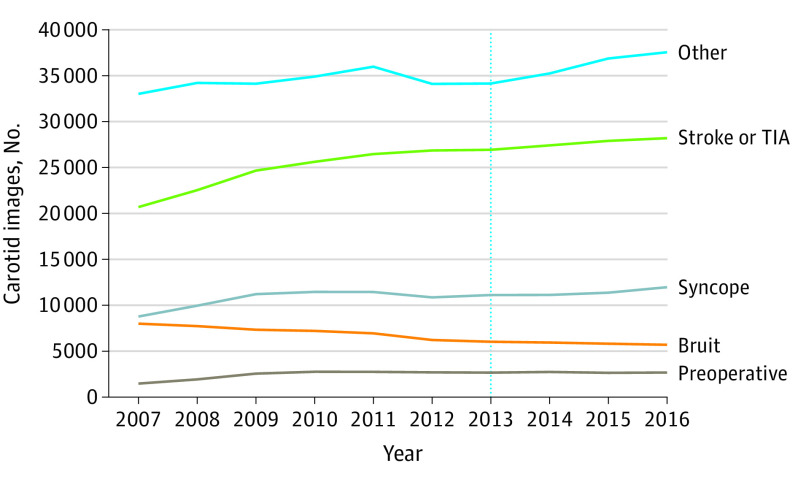
Annual Number of Carotid Imaging Performed in the Veterans Health Administration From 2007 to 2016 Vertical dotted line indicates release of the Choosing Wisely Guidelines; TIA, transient ischemia attack.

Nearly one-quarter of carotid images were ordered for low-value indications (201 467 images [24.9%]), while 257 369 images (31.8%) were performed for stroke workup, and 350 235 images (43.3%) were performed for other indications (Figure 1). Most images for low-value indications were performed for syncope (109 400 images [13.5%]), followed by for carotid bruit (67 064 [8.2%]), or for preoperative evaluation (25 032 [3.1%]). The annual number of images ordered for syncope increased from 8790 images in 2007 to 11 978 images in 2016 (36.3% increase). The annual number of images for preoperative evaluation increased from 1488 images in 2007 to a peak of 2776 images in 2010 (86.6% increase) and was then stable. The annual number of images for carotid bruit declined throughout the study period from 8014 images in 2007 to 5707 images in 2016.

### Trends in Carotid Imaging From 2006 to 2017

**Figure 2.**
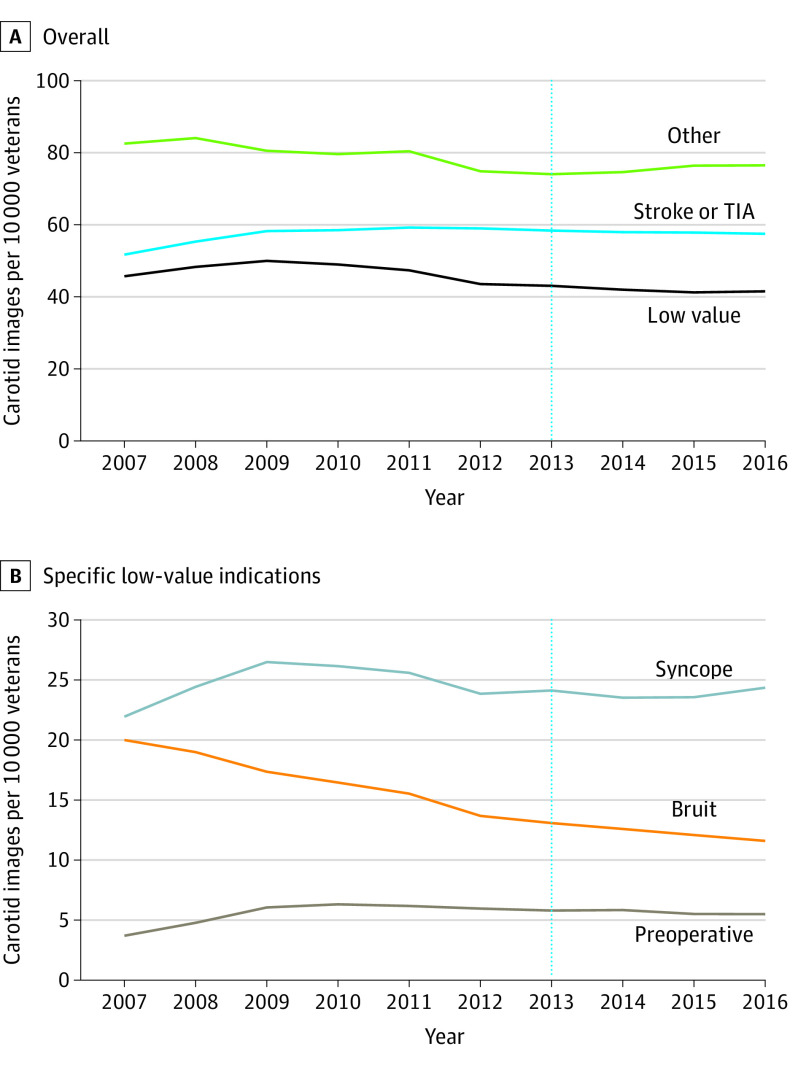
Rates of Overall and Low-Value Carotid Imaging From 2007 to 2016 Vertical dotted line indicates release of the Choosing Wisely Guidelines; TIA, transient ischemia attack.

For overall carotid imaging, there was no temporal trend before Choosing Wisely recommendations (annual rate of change, −0.2 [95% CI, −2.8 to 2.4] images per 10 000 veterans) and no immediate level change in the year of Choosing Wisely recommendation release (Table 2). However, there was a temporal increase in imaging in the post–Choosing Wisely period (annual rate of change, 1.8 [95% CI, −1.0 to 2.7] images per 10 000 veterans). There was no change in imaging for stroke workup and a statistically significant decrease in low-value carotid imaging and imaging for other indications (Figure 2A).

**Table 2.** Time Series Analysis of Carotid Imaging From 2007 to 2016

Indication	Carotid images, No. per 10 000 veterans per y (95% CI)				
	2007	Annual rate of change before Choosing Wisely	2013 Change in level	Annual rate of change after Choosing Wisely	Change in rate of imaging^a^
Overall	110.3 (103.7 to 116.8)	−0.2 (−2.8 to 2.4)	−4.9 (−16.0 to 6.1)	1.8 (1.0 to 2.7)	2.0 (−0.8 to 4.8)
Low value					
Bruit	13.8 (13.6 to 14.0)	−0.8 (−0.9 to −0.7)	0.2 (−0.4 to 0.8)	−0.3 (−0.3 to −0.2)	0.5 (0.4 to 0.6)
Preoperative	3.0 (2.1 to 3.9)	0.3 (0.03 to 0.6)	−0.8 (−2.0 to 0.3)	−0.1 (−0.1 to <−0.1)	−0.4 (−0.8 to −0.1)
Syncope	16.4 (13.9 to 18.8)	0.3 (−0.5 to 1.2)	−1.6 (−4.8 to 1.6)	0.2 (−0.1 to 0.6)	−0.1 (−1.0 to 0.8)

^a^ Compared with pre–Choosing Wisely trend.

Carotid imaging for syncope demonstrated no significant temporal trends overall, before (annual rate of change, 0.3 [95% CI, −0.5 to 1.2] images per 10 000 veterans) or after Choosing Wisely release (annual rate of change, 0.2 [95% CI, −0.1 to 0.6] images per 10 000 veterans) (Figure 2B and Table 2).

Imaging for carotid bruits declined across the study period (Figure 2B) and was declining prior to Choosing Wisely recommendation release (annual rate of change, −0.8 [95% CI −0.9 to −0.7] images per 10 000 veterans) (Table 2). After Choosing Wisely, there was a continued but less steep decline imaging in the post–Choosing Wisely period (annual rate of change, −0.3 [95% CI, −0.3 to −0.2] images per 10 000 veterans), resulting in a relative change in rate comparing after the trend with before the trend 0.5 (95% CI, 0.4 to 0.6) images per 10 000 veterans.

There was no significant change in preoperative carotid imaging (Figure 2B). Preoperative carotid imaging was increasing prior to Choosing Wisely recommendations (annual rate of change, 0.3 [95% CI, 0.03 to 0.6] images per 10 000 veterans) (Table 2). After the release of Choosing Wisely recommendations, there was no immediate level change in carotid imaging rates, but there was a temporal decrease in preoperative imaging in the post–Choosing Wisely period of −0.1 (95% CI, −0.1 to <−0.1) images per 10 000 veterans, resulting in a relative change in rate overall of −0.4 (95% CI, −0.8 to −0.1) images per 10 000 veterans.

### Carotid Procedures Following Carotid Imaging

**Figure 3.**
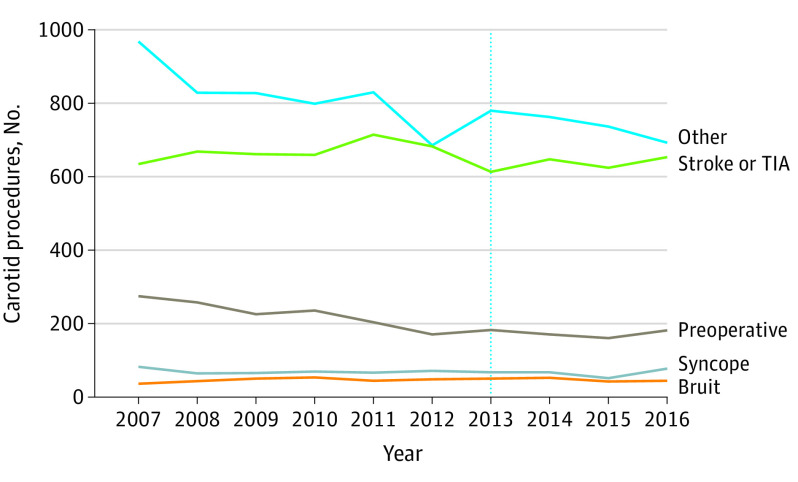
Trends in Carotid Procedures in the Veterans Health Administration Stratified by Indication of Preceding Carotid Image From 2007 to 2016 Vertical dotted line indicates release of the Choosing Wisely Guidelines; TIA, transient ischemia attack.

A total of 755 648 carotid images met criteria for carotid procedure analysis, of which 17 686 (2.3%) were followed by a carotid procedure within 6 months. Only 6555 carotid procedures (37.1%) were preceded by an imaging test ordered for stroke workup. The remaining procedures were preceded by an imaging test ordered for a low-value indication (3228 images [18.3%]) or other indications (7903 images [44.7%]) (Figure 3). The annual number of carotid procedures declined from 1996 procedures in 2007 to 1650 procedures in 2016, but the proportion performed after low-value imaging did not change significantly (395 procedures [19.8%] in 2007 vs 305 procedures [18.5%] in 2016; *P* = .07).

## Discussion

This serial cross-sectional study found that rates of overall carotid imaging in the national VHA health system remained stable between 2007 and 2016 and there was a small decline in low-value imaging driven by a decline in imaging for carotid bruits, which predated Choosing Wisely recommendations. We also observed a modest increase in imaging for syncope, despite Choosing Wisely recommendations, and a reversal of trends in preoperative imaging following Choosing Wisely recommendations, although the absolute magnitude of decline in imaging rates was small. These findings suggest that efforts to reduce low-value testing require more than public awareness campaigns.

The first key to successful efforts to reduce low-value testing is identifying tests with a high rate of overuse.^6,7,8,9^ However, baseline rates of testing that are targeted by Choosing Wisely recommendations are frequently unknown, particularly for recommendations targeting specific indications that may not be identifiable from administrative data. Thus, our finding that more than 200 000 carotid images ordered in the VHA health system during the study were for low-value indications demonstrates that the potential impact of reducing low-value carotid imaging is substantial. Priorities for initiatives targeting overuse should also focus on testing with significant costs and risk for downstream patient harms.^6,31^ Our finding that more than 3000 carotid revascularization procedures were performed following carotid imaging testing for low-value indications suggests that successful efforts to reduce low-value imaging may result in both the reduction of the direct costs of imaging and the downstream utilization of invasive procedures with uncertain benefits.

Our findings build on earlier studies examining trends following Choosing Wisely recommendations and overcome 2 key limitations of prior studies. First, prior studies have largely been limited to studying the period immediately following the recommendations release, thus delayed uptake of recommendations may have not been captured.^10,11^ Similar to these early studies, we found no immediate declines in low-value testing following the Choosing Wisely recommendations release and additionally found no substantial differences in testing during a 3-year follow-up period. Second, prior national studies of low-value testing have largely relied on administrative claims, which lack the ability to directly identify indications for testing.^10,22,23^ To overcome this limitation, we examined the indications for imaging entered by clinicians at the time of test ordering, using a novel text lexicon search strategy that allowed us to characterize overall and indication-specific imaging trends. Notably, we found that more than 40% of images were ordered for indications other than stroke workup or for low-value indications. This finding is consistent with prior literature indicating that there are many reasons physicians order carotid imaging, most of which are of uncertain clinical significance.^14^

While Choosing Wisely lists may include testing largely ordered by different specialties,^7^ in the case of carotid imaging, low-value indications were targeted by multiple specialties that are likely to order imaging: the Academy of Family Physicians,^17^ American Academy of Neurology,^19^ and the Society of Thoracic Surgeons.^20^ However, carotid images may also be ordered by other clinicians who may not be aware of these recommendations, including primary care clinicians (eg, general internists, nurse practitioners, or physician assistants), acute care clinicians (eg, hospitalists and emergency medicine physicians), and other surgical specialists. In the case of imaging for carotid bruits, we observed a decline in imaging over the period that predated the Choosing Wisely recommendations and may be associated with the 2007 USPSTF recommendations against carotid screening.^18^ Unlike Choosing Wisely lists, these recommendations are not specialty-specific and thus may have a wider impact.

To achieve success in reducing low-value carotid imaging, targeted interventions are needed. Quality improvement initiatives, including clinical decision support tools, clinician education, and direct feedback to ordering clinicians, have demonstrated promise in single-center and health system studies of other low-value testing.^32,33^ For imaging tests that may be ordered for different low-value indications, the target population for interventions may need to be adapted for each indication. Reducing low-value carotid imaging for acute conditions, such as syncope, likely requires clinician-focused interventions that span ambulatory, emergency department, and inpatient care settings. Whereas reducing low-value carotid screening tests requires both clinician- and patient-focused interventions, as patients may request low-value screening, driven by advertising from direct-to-consumer screening companies^34,35^ or receipt of prior testing.

Alternatively, interventions aimed at modifying ordering systems might address testing for multiple indications by either restricting the permissible indications for ordering carotid imaging or by providing clinical decision aids that inform clinicians of which indications may be of low value. Additionally, interventions may be more effective if they target the clinicians performing testing (eg, radiology and vascular departments) in addition to the ordering clinicians. An imaging stewardship model in which ordering and performing clinicians discuss the merits of testing for specific patients^36^ would allow for collaborative decision-making and education on the value of certain tests and build on well-accepted and successful antibiotic stewardship program models.^37^

### Limitations

Our study has several limitations. First, our study took place in the national VHA health system, which serves a unique and primarily male patient population with a high comorbidity burden, thus overall imaging rates may not reflect the wider US population. Second, the free-text fields entered by ordering clinicians that we examined to identify indications for imaging were only available for carotid images performed by VHA radiology departments and not those performed by vascular laboratories. Vascular laboratories record study results in progress notes and do not include easily identifiable information on the indication for testing. Thus, our study is limited to this subset of images, which accounts for approximately three-quarters of all carotid images performed in the VHA health system. Third, our analysis of carotid procedures following imaging is likely conservative, as it is possible that after a carotid image, some patients may have received a carotid procedure outside the VHA health system owing to patient preference or service availability. Fourth, we were not able to determine the specialty of the ordering clinician. Fifth, although our study identified indications for testing using a newly validated text lexicon approach that provides improved specificity for identifying indications for testing compared with prior studies relying on administrative claims, this approach was found to have only modest sensitivity for preoperative testing.

## Conclusions

The findings of this serial cross-sectional study suggest that the release of Choosing Wisely recommendations from 3 professional societies were not associated with a substantial reduction in low-value carotid imaging in the national VHA health system. To reduce low-value testing and utilization cascades, direct interventions targeting ordering clinicians are needed to augment the impact of public awareness campaigns.

## Notes

Supplement. **eAppendix.** Text Lexicon Development **eTable.** Codes for Carotid Procedures
