# Supplementary material for: Trends in Low-Value Carotid Imaging in the Veterans Health Administration From 2007 to 2016
Source: JAMA Netw Open. 2020 Sep 4;3(9):e2015250. doi: 10.1001/jamanetworkopen.2020.15250 (PMC7489844; doi:10.1001/jamanetworkopen.2020.15250)
Supplement: Supplement. — eAppendix. Text Lexicon Development eTable. Codes for Carotid Procedures [file jamanetwopen-e2015250-s001.pdf]

Supplementary Online Content

Anderson TS, Leonard S, Zhang AJ, et al. Trends in low-value carotid imaging in the Veterans Health Administration from 2007 to 2016. *JAMA Netw Open*. 2020;3(9):e2015250. doi:10.1001/jamanetworkopen.2020.15250

eAppendix. Text Lexicon Development
eTable. Codes for Carotid Procedures

This supplementary material has been provided by the authors to give readers additional information about their work.

© 2020 Anderson TS et al. *JAMA Network Open.*

eAppendix. Text Lexicon Development

When ordering a diagnostic testing in the VHA health system, the ordering clinician must complete documentation in two separate fields: noting the reason for the study (Study Reason field) and any relevant clinical history (Clinical History field). These two free text fields provide rich clinical documentation but are unstructured, thus we developed a text lexicon search strategy based on key clinical terms in order to identify the primary indications for imaging. The initial text search strategy was developed by two clinicians (TSA and SK) based upon common key words for each indication category of interest and included the following terms: *preoperative, clearance, syncope, presyncope, dizziness, lightheadedness, orthostatic hypotension, bruit, stroke, transient ischemic attack, and TIA.*

Initially search terms were applied to the “Study Reason” field to categorize carotid images into one of five categories: syncope, preoperative evaluation, carotid bruit, stroke/TIA work up, or other (if no search terms were identified). Next, a random sample of 200 carotid images from each category (1000 images total) were selected for chart review. A research assistant trained in clinical data abstraction (SJZ) who was blinded to the initial lexicon classification, then reviewed both the “Study Reason"” and “Clinical History” free text fields for each image and classified them into the five categories. This chart review served as the reference standard for further iterations of the lexicon. The test characteristics of the initial algorithm are displayed below:

	**Sensitivity**	**Specificity**
Stroke	72.7 (66.2 to 78.6)	93.9 (92.0 to 95.5)
Syncope	84.9 (79.5 to 89.3)	98.8 (97.8 to 99.5)
Bruit	94.9 (90.9 to 97.6)	98.5 (97.4 to 99.2)
Preoperative	86.7 (78.4 to 82.7)	87.3 (85.9 to 89.4)
Other	55.2 (49.4 to 61.0)	94.9 (93.0 to 96.4)

While the initial text lexicon search terms were fairly specific, they lacked sensitivity, thus misclassified images were reviewed by the study team to identify common misspellings (e.g. brut rather than bruit), abbreviations (e.g. preop rather than preoperative), and related symptoms (e.g. fainting rather than syncope). The initial search for preoperative indications had lower specificity, due to misclassification of indications which included information on “history of surgery” rather than preparation for surgery. Thus, to improve the accuracy for the search strategy the final lexicon was applied to both the “Study Reason"” and “Clinical History”. The test characteristics of the final lexicon search strategy is provided below:

	**Sensitivity**	**Specificity**	**Prevalence**	**True Positives**	**False Positives**	**True Negatives**	**False Negatives**
Stroke	89.5<br>(84.5 to 99.3)	91.7<br>(89.5 to 93.5)	209	187	66	728	22
Syncope	92.4<br>(88.2 to 95.5)	99.4<br>(98.5 to 99.8)	225	208	5	770	17
Bruit	94.4<br>(90.3 to 97.2)	99.1<br>(98.2 to 99.6)	198	187	7	795	11
Preop	77.6<br>(68.0 to 85.4)	99.0<br>(98.1 to 99.5)	98	76	9	893	22
Other	81.2<br>(77.0 to 86.0)	91.2<br>(88.8 to 93.2)	297	243	62	641	54

© 2020 Anderson TS et al. *JAMA Network Open.*

eTable. Codes for Carotid Procedures

	**Specific Codes**
CPT	35301, 37215, 37216
ICD-9	38.12, 00.61, 00.63
ICD-10	03CH*, 03CJ*, 03CK*, 03CL*, 037H*, 037J*, 037K*, 037L*

© 2020 Anderson TS et al. *JAMA Network Open.*
